# Data Sharing in a Humanitarian Organization: The Experience of Médecins Sans Frontières

**DOI:** 10.1371/journal.pmed.1001562

**Published:** 2013-12-10

**Authors:** Unni Karunakara

**Affiliations:** Médecins Sans Frontières, Geneva, Switzerland

## Abstract

Unni Karunakara and colleagues discuss how Médecins Sans Frontières decided to adopt a data sharing policy for routinely collected clinical and research data in humanitarian settings and its aspirations to create a truly open data set with the first step being managed access.

*Please see later in the article for the Editors' Summary*

**Summary Points**

- Public health crises such as the spread of drug-resistant tuberculosis highlight the need for improved sharing of data. For humanitarian organizations, there is a lack of guidance on the practical aspects of making such data available.
- In 2012 the medical humanitarian organization Médecins Sans Frontières (MSF) decided to adopt a data sharing policy for routinely collected clinical and research data. Here we describe how this policy was developed, the principles underlying it, and the practical measures taken to facilitate data sharing.
- The MSF policy builds on the principles of ethical, equitable, and efficient data sharing to include aspects relevant for an international humanitarian organization, in particular concerning highly sensitive data (non-maleficence), benefit sharing (social benefit), and intellectual property (open access).
- There are aspirations to create a truly open dataset, but the initial aim is to enable data sharing via a managed access procedure so that security, legal, and ethical concerns can be addressed.

## Introduction

Open data and data sharing are essential for maximizing the benefits that can be obtained from institutional and research datasets [1]. In 2012, the medical humanitarian organization Médecins Sans Frontières (MSF) decided to adopt a data sharing policy for routinely collected clinical and research data (http://www.msf.org.uk/msf-data-sharing). Here we describe the policy's principles, practicalities, and development process. We hope this paper will encourage and help other humanitarian and nongovernmental organizations to share their data with public health researchers for the benefit of the populations with which they work.

### The Growth of Open Data

Initiatives to promote the sharing of data generated by research activities have been led by foundations such as the Wellcome Trust and other signatories to the Full Joint Statement by Funders of Health Research [2], the creation of large open databases such as Dryad [3], and journal and publisher initiatives [4]–[7]. However, practical and systemic limitations have limited real data sharing across medical and clinical research [8] and routinely collected clinical data [9]. Although much discussion has taken place around data sharing (Theodora Bloom, personal communication), concrete actions and a positive willingness to share data have been less common.

### Datasets Collected in Humanitarian Situations

Public health crises, such as the spread of drug-resistant tuberculosis [10] and the 2002 severe acute respiratory syndrome (SARS) outbreak [11], highlight the need for sharing data; a case has been made that data sharing is an ethical duty in such contexts [12]. For humanitarian organizations, there is a lack of guidance on how and what sort of data can and should be shared, and especially on the practical aspects of making such data available while considering the sensitivities involved in datasets collected in contexts of humanitarian action.

### MSF and Data Sharing

MSF and Epicentre, its research affiliate (http://www.epicentre.msf.org/en), place a high value on monitoring and documenting MSF's medical interventions to improve their quality, resulting in a large amount of routinely collected data. In addition, MSF conducts a substantial amount of operational research with patient groups and diseases commonly neglected in international research agendas [13],[14]. MSF recognizes its responsibility to share and disseminate this knowledge. As a first step in meeting this responsibility, MSF established an institutional repository for its research publications (http://fieldresearch.msf.org/msf/) in 2008, and more recently has introduced a scientific publication policy that prioritizes open access, and is working on a policy for online sharing of research protocols.

### Development of the MSF Data Sharing Policy

Until 2012, decisions to share MSF data were made on a case-by-case basis on request. Recognizing the problems inherent in this informal approach, MSF developed a proactive data sharing policy in the hope of boosting data sharing while ensuring that ethical and legal obligations were met (Box 1). The principles in the Full Joint Statement by Funders of Health Research [2] were the starting point for the MSF policy, namely, that data should be shared in a manner that is ethical, equitable, and efficient. MSF consulted with the Wellcome Trust and the MSF Ethics Review Board [15] to adapt and expand these principles to include ones specific for MSF concerning highly sensitive data, benefit sharing, and intellectual property. The policy was drafted using a template from the UK National Cancer Research Institute [16].

**Box 1. Issues Requiring Ethical Review**

The independent MSF Ethics Review Board was created to ensure that ethical oversight is available for issues that could arise from a humanitarian organization providing care and also requesting participation in research. In determining the procedures for our data sharing policy, two situations were identified as needing ethical review.

One was the inclusion of personal (identifiable) data and/or human samples (with adequate consent), given the high sensitivity of MSF contexts and—generally speaking—of human samples. Sharing of personal data or human samples potentially entails risk in terms of the perception by MSF patients and authorities in countries of operation that MSF is carrying out research under the guise of medical care. It was decided not to exclude outright the secondary use of personal (identifiable) data and/or human samples—as some of these data can be of considerable value to research that promotes health benefits. Where personal data are included in a dataset, ethical review is required.

The second situation was the use of nonidentifiable research data outside of original consent agreements, which some MSF Ethics Review Board members felt should not be authorized. However, there will be rare cases of research data collected prior to the data sharing policy being created that have significant value for communities, particularly those relating to neglected diseases, where a case can be made that the benefits of sharing such data outweigh the potential harms. After considerable debate, the use of nonidentifiable research data outside of original consent agreements was accepted if MSF tries to return to study participants to expand their original consent or, failing that, is able to secure consent from the community where the study took place. Use of data outside of original consent will always require ethical review.

## Vision and Principles

MSF commits to share and disseminate health data from its programs and research in an open, timely, and transparent manner in order to promote health benefits for populations while respecting ethical and legal obligations towards patients, research participants, and their communities. MSF will work towards maximizing the availability of health data of wider interest to public health researchers with as few restrictions as possible, while respecting the principles outlined in Box 2. Practically, these ambitions will be achieved by creating an online data collection.

**Box 2. Principles Underlying Data Sharing in MSF**

**Ethics:** MSF data sharing will abide by the following ethical principles:

- **Medical confidentiality** is fully respected.
- The **privacy and dignity of individuals** and communities are not jeopardized.
- **Collaborative partnerships** are undertaken in line with MSF's Ethical Framework for Medical Research; recipients of MSF datasets will engage, wherever possible, with the local research community and the local community where the MSF dataset originates.

**Equity:** MSF data sharing will recognize and balance the needs of practitioners or researchers who generate and use health data, other analysts who may want to reuse such data, and communities and funders who expect health benefits to arise from research.

**Efficiency:** MSF data sharing will improve the quality and value of the delivery of health care, and increase its contribution to improving public health. Approaches should be proportionate and build on existing practice and reduce unnecessary duplication and competition.

**Non-maleficence:** Data sharing shall not put at risk, or be used against, the interests of MSF patients, MSF research participants, MSF employees, or MSF organizations for political reasons, financial gain, or any other reasons.

**Social benefit:** First, to promote health benefits to the greater population, data sharing should bring health benefits to individuals and communities outside of those in which the data were collected. Second, to prioritize local benefit sharing, data sharing will prioritize data of benefit to the local communities where the data were collected, as well as to patients and communities similar to those in which MSF works, in particular marginalized or neglected populations. Notwithstanding this, there is a recognition that benefit sharing can be with a wider community of individuals, and will not always result in benefits to the local community.

**Open access:** Recipients of MSF datasets shall strive to avoid prohibitively costly approaches, restrictive intellectual property strategies, or other approaches that may inhibit or delay the use of the results of their research to the benefit of low- and middle-income countries. In particular, they shall put forth their best efforts to avoid anything that could seriously limit follow-up research and/or development and/or equitable and affordable access to potential final product(s) by end users in such countries. Recipients shall not seek any intellectual property rights of any kind with respect to results generated by or arising out of the use of MSF datasets without prior written consent.

### Principles Developed for the MSF Data Sharing Policy

#### Non-maleficence

MSF projects are often located where there is political or ethnic violence, or where certain disease diagnoses are associated with government restrictions or potentially dangerous consequences. The overriding imperative for MSF is to ensure that patients are not harmed or compromised. Thus, caution is needed when handling potentially sensitive data. Sensitive data are defined as any subset of information that can be misused against the interests of the individuals whose data are included in the dataset or against MSF, or that put either individuals or MSF at risk for political, financial, or other reasons (Box 3). In determining the eligibility of datasets for sharing, MSF must consider their potential sensitivity and ensure that appropriate safeguards are in place. Should safeguards not be appropriate or sufficient, MSF may decide that datasets are not be eligible for sharing.

**Box 3. Sensitive Data**

**Data considered sensitive by MSF:**

- Any data from which an implication of criminal conduct could be drawn and/or that can put MSF patients or research participants at serious risk (including death). This includes data on violence-related medical activities, particularly, but not exclusively, in contexts of conflicts: (1) any data related to violence—such as bullet wounds—and (2) any data related to sexual violence.
- Data collected from MSF activities in prisons or any situation that are related to or can result in detention or deprivation of liberty (including in certain refugee or displaced person settings).
- Certain data variables such as those that could indirectly imply, truly or not, racial or ethnic origin, or political or religious opinions (for example, the origin or the location of the patient/participant).
- Data related to sicknesses with an obligation to adhere to treatment.

**Data considered potentially sensitive by MSF (non-exhaustive):**

- Data that can put patients/participants at risk of stigma, discrimination, or criminal sanction (including, in certain countries or populations, HIV and tuberculosis data).
- Data on sicknesses or epidemic outbreaks.

#### Social benefit

MSF will prioritize data sharing requests that are of benefit to the local communities where the data were collected, as well as to patients and communities similar to those in which MSF works, in particular marginalized or neglected populations. Notwithstanding this, there is a recognition that benefit sharing can be with a wider community of individuals, and will not always result in benefits to the local community.

#### Open access

In 1999, MSF launched the Access Campaign to push for access to, and the development of, medicines, diagnostic tests, and vaccines for patients in MSF programs and beyond. Research developed as a result of data shared by MSF should remain consistent with such aims, with results and end products being accessible (and affordable) in low- and middle-income countries. In light of the potential public health benefits of releasing results immediately and without restrictions, publication of results should be consistent with the MSF scientific publishing policy, which prioritizes open access.

Access to MSF datasets will be granted only if the recipients of data agree not to seek intellectual property rights of any kind, without MSF giving specific and prior consent. In addition, recipients must avoid actions that render the results of their research, such as publications or medical products, unavailable or unaffordable for the populations of low- and middle-income countries.

### What Data Will Be Included in the Data Collection?

The policy applies to all health data generated in MSF programs or sites, where MSF acts as a custodian for such data. It includes data generated from health information systems, patient records, surveillance activities, quality control activities, surveys, research, and patients' or research participants' human biological material. While the scope of the policy is purposely broad, there is no ambition to share data simply for the sake of sharing. Only data whose dissemination is judged to have the potential to lead to greater health benefits for populations will be shared (Box 2). Practically, this decision-making process will be implemented through a procedure whereby MSF data judged to have a substantial public health benefit are eligible to be proposed by any MSF or Epicentre staff for inclusion in the online collection. The decision to include data will be guided by the vision and principles of the data sharing policy, and data should not be unreasonably withheld. Approval for data sharing may have to be sought from other involved partners where preexisting contracts or memorandums of understanding limit data sharing.

Data initially proposed for inclusion include records of HIV treatment and care, treatment for drug-resistant tuberculosis and human African trypanosomiasis, and a database of nutritional surveys. Research data will be added as they become available.

### Managed Access Procedure

#### Who can access the data collection?

Access to the data collection will be open to all appropriately qualified researchers from academia, charitable organizations, and private companies, such as drug companies. MSF defines an appropriately qualified researcher as someone who has authored relevant peer-reviewed articles, and who is still working in the relevant specialty [17]. We will positively consider all applications from researchers from countries and communities in which we work and, in particular, from where the specific datasets requested originated.

#### How will access be managed?

We intend to post some datasets in an open repository, but as a first step to gain experience with data sharing, managed access will be the default means of sharing data. A high proportion of data generated by MSF is considered sensitive, thereby requiring a higher level of oversight. The stringency of the managed access procedure will be proportionate to the risks associated with MSF datasets, and must not unduly restrict or delay access.

#### Costs

Most of MSF's funding comes from individual private donors who wish to support medical humanitarian assistance. Thus, MSF has chosen to implement data sharing as a cost-neutral exercise. Recipients of data will be required to cover the costs of retrieving, processing, and dispatching MSF datasets. If applicants for data sharing do not have sufficient financial means to cover such fees, exceptions can be made.

## Challenges

### Data Collection and Protection

The MSF data sharing policy is based on MSF's organizational commitment to improving the ethical collection and protection of data in our programs. The nature of humanitarian contexts can make this challenging, particularly in terms of the ability to obtain informed consent for data collection. Ensuring the privacy and confidentiality of the data collected also requires specific attention. For example, tissue samples have specific ethical issues attached to their collection, use, and dissemination. In MSF, material transfer agreements are now signed with external laboratories that provide advanced testing for our patients. This ensures that samples are not used without consent for purposes other than those requested by MSF clinicians, and that they are disposed of correctly.

### Ensuring MSF Staff Share Data

The data sharing policy is aspirational and will rely on political engagement to ensure compliance. This is challenging because the scope of the policy with regards to routinely collected data means that the participation of MSF staff in program and headquarter offices is required, as well as that of staff involved in research, who may already appreciate the value of sharing research-generated datasets. Data sharing will be facilitated with standard templates to support development of data sharing plans and proposals.

### Ensuring Inclusion of Data Sharing in Research Proposals

At the research proposal stage, if the research is likely to generate data outputs valuable for the wider public health community, MSF researchers should develop a data management and sharing plan that includes consideration of the resources required. The inclusion of a broad consent in research proposals will be considered where there is evidence of a clear potential for the greater public good and if risks are limited. Broad consent is usually granted ethics approval under the conditions that personal information is handled safely and that the donors of biological samples are granted the right to withdraw consent.

### Data Quality

The value of the data sharing policy will rely on good practices in data collection, use, and management [18]. As an organization focused on providing emergency assistance, creating and maintaining datasets to a high standard is a continual challenge. Organizationally, there is commitment to strengthening standards and an expectation that data sharing itself will strengthen this process with a consistent and positive engagement with researchers and dataset managers. In addition, MSF will prioritize information technology solutions that facilitate data sharing.

### Data Preservation

Preserving and protecting data from corruption or obsolescence of software is a serious concern with open data and data sharing. Digital Science offers a research data archiving service via Figshare and notes the safeguards needed to ensure the preservation and security of data [19]. As the MSF data sharing database grows, data preservation may require innovative thinking to ensure its security.

## The Way Forward

MSF's core mission is to respond to medical humanitarian crises. This priority makes it quite unlike the large research-oriented organizations and funders that have pioneered data sharing. MSF's data sharing policy will test the ability of the organization to protect the vulnerable population it serves while contributing to health research to ultimately benefit the communities and patients from which the data were gathered.

## Abbreviations

MSF: Médecins Sans Frontières
